# Identification of Marrubiin as a Cathepsin C Inhibitor for Treating Rheumatoid Arthritis

**DOI:** 10.3390/molecules30214170

**Published:** 2025-10-23

**Authors:** Fei-Long Zhou, Yu Zhang, Cui Chang, Da-Xing Shi, Xing Chen, Xin-Hua Liu, Xiao-Bao Shen

**Affiliations:** 1 Medical School, Fuyang Normal University, Fuyang 236037, China; 2025560128@ahmu.edu.cn; 2 Inflammation and Immune Mediated Diseases Laboratory of Anhui Province, School of Pharmacy, Anhui Medical University, Hefei 230032, China; 2445011042@stu.ahmu.edu.cn (Y.Z.); 2345010981@stu.ahmu.edu.cn (C.C.); 2345010818@stu.ahmu.edu.cn (D.-X.S.)

**Keywords:** cathepsin C, marrubiin, arthritis, natural product, anti-inflammatory

## Abstract

Cathepsin C (CTSC) mediates neutrophil serine protease (NSP) maturation, contributing to inflammatory cascades, making it a key therapeutic target. In this study, through large-scale screening of a natural product library, marrubiin, a diterpenoid lactone compound, was identified as a potent CTSC inhibitor, which holds potential value in the treatment of inflammatory diseases. It inhibited human recombinant CTSC (IC_50_ = 57.5 nM) and intracellular CTSC (IC_50_ = 51.6 nM) with acceptable cytotoxicity, and reduced the activity and protein levels of downstream NSPs in vitro. Functionally, marrubiin inhibited lipopolysaccharide-induced nitric oxide release and regulated the levels of cytokines and chemokines. Docking result predicted marrubiin may achieve CTSC activity inhibition by using lactone structure as a covalent unit to target Cys234. In vivo study indicated that high-dose marrubiin (IC_50_ = 30 mg/kg) reduced CTSC and NSPs activities in blood and bone marrow in mice without toxicity, and its efficacy was comparable to that of positive compound AZD7986. In the adjuvant-induced arthritis model, high-dose marrubiin (IC_50_ = 60 mg/kg) exerted a therapeutic effect by reducing the activities of CTSC and NSPs. These findings indicated marrubiin is a promising natural CTSC inhibitor, which can be used for the treatment of neutrophil-related inflammatory diseases.

## 1. Introduction

**Figure 1:**
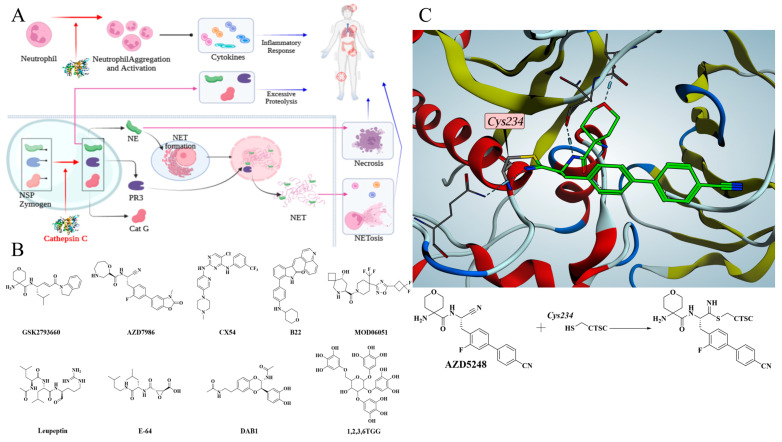
(**A**) CTSC participates in inflammatory cascade through multiple pathways and promotes disease progression. (**B**) Representative CTSC Inhibitors from synthetic molecules and natural products. (**C**) Co-crystal structure reveals that the covalent compound AZD5248 inhibits CTSC activity by binding to Cys234.

Cathepsin C (CTSC) is a lysosomal cysteine peptidase belonging to the papain family [1]. Its core function is to mediate the processing and maturation of granule-associated serine proteases by highly specifically removing dipeptidyl units from the N-terminus of peptides or proteins [2]. CTSC is involved in the processing of various serine proteases, and plays an irreplaceable role in the final maturation of neutrophil serine proteases (NSPs) [3,4,5,6,7]. NSPs, with neutrophil elastase (NE), proteinase 3 (PR3), and cathepsin G (Cat G) as core components, are synthesized as zymogens at the promyelocyte stage in the bone marrow, stored in primary granules, and converted to active form after the N-terminal dipeptide is cleaved by CTSC [2,7]. Under pathological conditions, activated NSPs are excessively released, leading to tissue damage and driving the occurrence of inflammatory cascade reactions [2,8,9]. Therefore, directly inhibiting CTSC activity to prevent NSP maturation has become an attractive strategy for inflammatory diseases (Figure 1A). Therefore, CTSC inhibitors have been developed for the treatment of non-cystic fibrosis bronchiectasis (NCFBE), chronic obstructive pulmonary disease (COPD), rheumatoid arthritis (RA), inflammatory bowel disease (IBD), and anti-neutrophil cytoplasmic antibody-associated vasculitis [10,11,12].

Currently, multiple molecules from pharmaceutical companies have entered clinical trials for the treatment of inflammatory diseases (Figure 1B) [13,14,15,16,17,18]. GSK2793660 from GlaxoSmithKline, characterized by an α,β-unsaturated enone as the covalent moiety, was the first small-molecule inhibitor to enter clinical trials but was eliminated due to failure to achieve the expected clinical benefits [13]. AZD5248, a cyano-based covalent inhibitor of CTSC, was discontinued despite its excellent in vitro activity, primarily due to its potential aortic binding toxicity in vivo [14]. AZD7986, as the follow-up modified product of AZD5248, is a successful small-molecule drug with favorable efficacy and safety [15]. The co-crystal structures of compounds and CTSC demonstrate that CTSC inhibitors form covalent bond with Cys234, the key amino acid residue, via covalent warhead structures, through which the activity of CTSC is inhibited (Figure 1C) [14]. Many researchers remain dedicated to developing novel CTSC inhibitors for treating various inflammatory diseases. Several non-covalent CTSC inhibitors, such as compounds CX54, B22, and MOD06051, have been reported for the treatment of lung injury, IBD, and neutrophil associated inflammatory diseases [19,20,21,22].

The isolation of natural products from natural resources has a long history, especially in application of anti-infection and anti-inflammatory therapies [23,24]. Multiple natural products have been reported to exhibit CTSC inhibitory effects [25,26,27]. Leupeptin, which uses an aldehyde group as warhead, and E-64, whose warhead is a propylene oxide group, are microbial secondary metabolites produced, respectively, by *Streptomyces roseus MB-26-AI* and *Aspergillus japonicus*. Both are broad-spectrum protease inhibitors that exhibit inhibitory activity against CTSC [1]. Anti-inflammatory evaluation results indicated that DAB1 isolated from *Oxya chinensis* is an effective anti-inflammatory agent by exerting CTSC inhibitory effect [28]. Notably, these natural products show moderate or low inhibitory activity against CTSC, often requiring high doses to achieve complete inhibition. 1,2,3,6-tetra-O-galloyl-β-d-glucose (β-1,2,3,6TGG), isolated from the *Galla chinensis*, exhibits potent inhibitory activity against CTSC, serving as a natural inhibitor for treating inflammatory diseases [29]. Identifying potential CTSC inhibitors from natural products and performing structural modification represent an efficient approach for developing novel drug to treat neutrophilic inflammatory diseases [30,31,32].

Diterpenoid lactone structures are characteristic natural product scaffolds with well-defined pharmacological activities, whose core framework is composed of 4 isoprene units (20 carbon atoms) and contains at least one lactone ring (-CO-O-) in the molecule [33,34]. This combination of “diterpene carbon skeleton + lactone ring” serves as the structural basis for their pharmacological activities [34]. Due to differences in structural details such as double bond positions, substituents, and the size of lactone rings, they exhibit diverse and potent pharmacological effects, including anti-inflammatory, anti-tumor, anti-viral, and immunomodulatory activities, thus acting as an important source of lead compounds for drug development [35,36,37]. Plants of *Marrubium* genus, commonly known as horehound, belong to the lamiaceae family and are frequently used as food flavorings and medicinal materials [38]. Marrubiin is a diterpenoid lactone compound present in lamiaceae plants, typically isolated from species such as *Lagopsis supina*. and *Marrubium vulgare* [39]. As a diterpene with a long history, it exhibits multiple bioactive effects including antioxidant, antifungal, immunomodulatory, cardioprotective, antidiabetic, and analgesic properties [40,41,42,43,44,45]. Numerous studies have been reported, which reveal the pharmacological potential of marrubiin from various dimensions and emphasize its important role in anti-inflammation [46]. Notably, other researchers have found that marrubiin inhibits carrageenan-induced peritoneal inflammation by preventing inflammatory cell infiltration and peritoneal mast cell degranulation [47]. Regrettably, there have been no clear research findings identifying the specific target through which marrubiin exerts its anti-inflammatory effects. In this study, through large-scale screening of a natural product library, marrubiin was confirmed as a CTSC inhibitor, and showed anti-arthritic activity in a adjuvant-induced arthritis (AIA) rat model.

## 2. Results

### 2.1. Screening Campaign for Putative Cathepsin C Inhibitors

**Figure 2:**
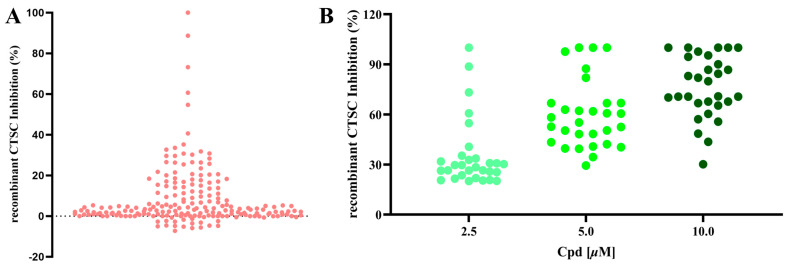
A large-scale screening campaign to identify CTSC inhibitors. (**A**) Large-scale screening of 200 natural products using the in vitro CTSC enzyme assay. Scatterplot of CTSC inhibition (%) in the presence of library compounds at 2.50 μM. (**B**) Confirmation of 30 hit compounds from the screening campaign, exemplified by three-point (2.5, 5, and 10 μM) dose–response inhibitions of CTSC in enzyme assay. Each group of tests was independently repeated at least 3 times.

**Figure 3:**
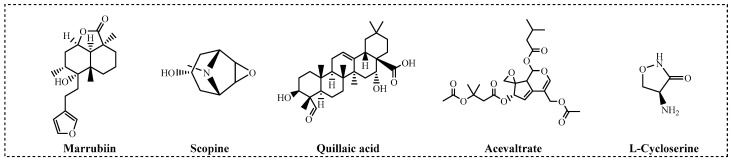
Natural products maintaining high-level inhibitory activity against CTSC.

To identify natural products with inhibitory effects on CTSC activity, we collected and constructed a molecular library containing 200 compounds, all of which were confirmed to be unmodified natural products. A large-scale screening of this library was conducted at a test concentration of 2.50 μM using a human recombinant CTSC isolated enzyme activity inhibition assay (Table S1). AZD7986, an artificially synthesized, highly potent and selective CTSC inhibitor, was selected as the positive control, with an IC_50_ value of 9.0 nM. 55 molecules were identified to exhibit varying degrees of inhibitory activity during the test (with a CTSC enzyme inhibition rate ≥ 10.0%) (Figure 2A). 30 molecules with an inhibition rate exceeding 20% were subjected to multi-concentration confirmation experiments (Figure 2B, Table S2) to further identify potential CTSC inhibitors. It is reassuring that 5 natural products, which were identified as effective CTSC inhibitors and will be used for further activity evaluation, still maintain a high level of inhibition on CTSC activity (with CTSC isolated enzyme inhibition rate ≥ 50.0% at 2.50 μM) (Figure 3).

### 2.2. Marrubiin as a Potent and Selective CTSC Inhibitor

To comprehensively evaluate the enzymatic inhibitory characteristics of five natural products, a 12-point dose–response curve for systematic testing was employed in this study (Table S3). Considering that compounds containing covalent modification groups may exhibit reduced intracellular activity and potential cytotoxicity due to metabolic instability and high reactivity, cell-based CTSC inhibition assay and cytotoxicity evaluation using mammalian cells were concurrently conducted (Table 1). Results from concentration gradient experiments revealed that the activity differences observed in single-concentration tests were further amplified. Scopine, a covalent inhibitor featuring an epoxypropane moiety and an α1-adrenergic receptor agonist [48], demonstrated CTSC inhibitory activity with consistency between cell-free enzymatic assays (IC_50_ = 134.8 nM) and intracellular enzyme inhibition (IC_50_ = 337.0 nM). In contrast, acevaltrate, a natural product with broad biological activities [49], retained CTSC inhibitory activity but was one order of magnitude less potent (IC_50_ = 1053.2 nM). Quillaic acid, characterized by an aldehyde-terminal structure [50], displayed potent CTSC inhibition in cell-free system (IC_50_ = 125.0 nM) but failed to inhibit substrate degradation in intracellular assays (IC_50_ > 5000 nM). l-cycloserine, a metabolite of *Mycobacterium tuberculosis* [51], exhibits potent inhibitory activity against recombinant human CTSC (IC_50_ = 1101.4 nM) but lacks intracellular activity (IC_50_ > 5000 nM). Of particular note is marrubiin, a polycyclic compound characterized by a lactone structure [39], which exhibits significant advantages in terms of enzyme activity (marrubiin IC_50_ = 57.5 nM). Cytotoxicity assessments indicated that none of the tested natural products significantly affected the viability of human histiocytic lymphoma U937 cells or mouse monocyte-macrophage leukemia RAW264.7 cells at the maximum concentration (10 μM). While marrubiin exhibited lower potency than the clinical candidate AZD7986 in cell-free assays, its intracellular inhibitory activity was comparable (marrubiin IC_50_ = 51.6 nM vs. AZD7986 IC_50_ = 23.5 nM), establishing it as the lead compound in this study.

Cathepsin selectivity profiling showed that marrubiin exhibited different levels of inhibitory activity against members of the cathepsin family. Specifically, it exerted only weak inhibitory effects on cathepsin B (CTSB) and cathepsin L (CTSL), while showing no inhibitory effects on cathepsin S (CTSS) and cathepsin K (CTSK) (Table 2). Furthermore, the inhibitory activity of marrubiin against key members of the dipeptidyl peptidase family was tested, and the results demonstrated that marrubiin had no inhibitory activity against any of the three dipeptidyl peptidases (Table 3). Finally, considering the need to rule out the possibility that marrubiin exerts its inhibitory effect on NSPs activity by inhibiting CTSC rather than directly inhibiting NSPs, the inhibitory activity of marrubiin against NSPs was tested. The results indicated that marrubiin also showed no inhibitory activity against any of the three NSPs (Table 4). Collectively, these results underscore marrubiin, a bioactive natural lactone isolated from *Marrubium vulgare*, as a promising candidate for further in vitro and in vivo efficacy evaluations.

### 2.3. Marrubiin Exerts Anti-Inflammatory Effect via Modulating Cytokine Levels

**Figure 4:**
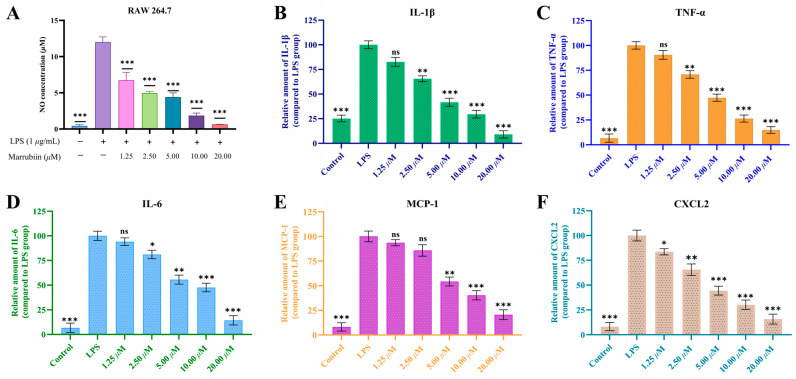
The dose-dependent inhibition of LPS-induced NO release (**A**), IL-1β (**B**), TNF-α (**C**), IL-6 (**D**), MCP-1 (**E**) and CXCL2 (**F**) secretion by RAW 264.7 macrophages after treatment with marrubiin for 24 h. NO inhibition was detected by Griess reagent. The cytokine levels were measured using ELISA kits. Each group of the aforementioned experiments was independently repeated at least three times. GraphPad Prism 10.0 software was used to process and analyze the experimental data by means of One-way analysis of variance. Statistical significance was considered: ns = no significant. * *p* < 0.05, ** *p* < 0.01 and *** *p* < 0.001 vs. LPS model group.

CTSC has been confirmed to regulate the levels of various inflammatory cytokines, represented by interleukin-1 beta (IL-1β), interleukin-6 (IL-6), tumor necrosis factor-alpha (TNF-α), monocyte chemoattractant protein-1 (MCP-1) and C-X-C motif chemokine ligand 2 (CXCL2), through direct or indirect pathways, and plays a core regulatory role in the progression of inflammatory diseases [30]. To evaluate the anti-inflammatory activity of marrubiin, its inhibitory effect on the release of nitric oxide (NO), an inflammatory mediator, and its regulatory effect on levels of various cytokines were detected. The result showed that within the safe administration concentration range, marrubiin exerted anti-inflammatory activity, with an IC_50_ of 0.83 μM for NO release, suggesting that it is an anti-inflammatory natural product (Figure 4A). Further research results indicated that LPS stimulation significantly upregulated the relative contents of various inflammatory factors (TNF-α, IL-6, IL-1β) and chemokines (MCP-1, CXCL2) in RAW 264.7 cells. After treatment with marrubiin, the relative levels of the above-mentioned cytokines were reduced to varying degrees, and this effect was significantly enhanced with the increase in marrubiin concentration (Figure 4B–F).

### 2.4. Marrubiin Enhances Protein Stability by Binding to Intracellular CTSC

**Figure 5:**
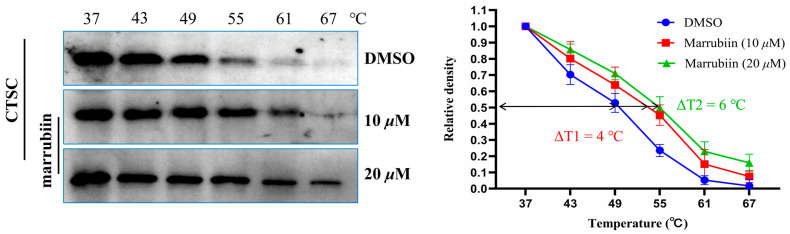
CETSA in U937 cell lysates treating with marrubiin. Each group of tests was independently repeated at least 3 times. GraphPad Prism 10.0 software was used to process and analyze the experimental data.

After confirming that marrubiin could inhibit CTSC activity and exert anti-inflammatory effect in cells, its binding to intracellular target was further evaluated by using cellular thermal shift assay (CETSA) [52]. U937 cells were equally divided into three groups and incubated with different concentrations of marrubiin solutions (10 μM, 20 μM) or an equal volume of dimethyl sulfoxide (DMSO) solution for 2 h to ensure sufficient binding between marrubiin and CTSC. Following treatment, cells were collected and lysed. After protein quantification, each cell lysate was divided into six aliquots and incubated at 37 °C, 43 °C, 49 °C, 55 °C, 61 °C, and 67 °C for 5 min. The relative density of CTSC was normalized to that of the unheated sample. By plotting the temperature-dependent curves of target protein remaining, the differences between experimental and control groups were compared. The ΔT value, defined as the temperature difference at which 50% of CTSC remained between the experimental and control groups, was calculated to evaluate the ability to enhance target protein thermal stability. Results showed that in DMSO control group, CTSC underwent significant degradation at 49 °C and was almost completely degraded at 61 °C. In contrast, treatment with marrubiin enhanced the thermal stability of CTSC in a concentration-dependent manner, with the temperatures at which significant degradation occurred increasing to approximately 61 °C and 67 °C at 10 μM and 20 μM, respectively. CETSA results indicated that marrubiin enhances the thermal stability of CTSC in a dose-dependent manner, and this effect becomes more pronounced with increasing temperature (Figure 5).

### 2.5. Docking Study of Marrubiin

**Figure 6:**
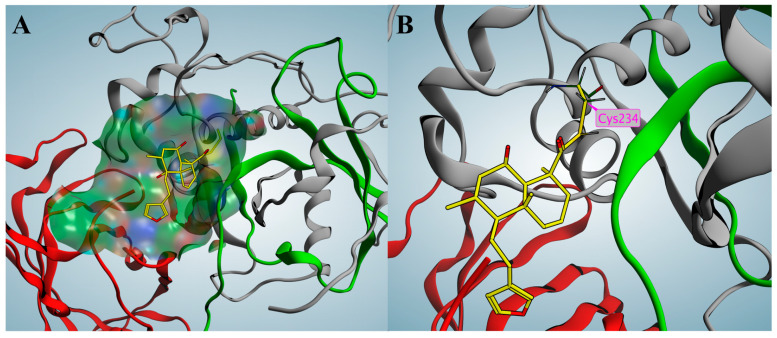
Covalent docking of marrubiin with CTSC. (**A**) Marrubiin marked in yellow occupies the active pocket; (**B**) Marrubiin forms a covalent bond with the key residue Cys234. The gray, green, and red ribbons represent the heavy chain, light chain, and exclusion domain, respectively.

To predict the potential binding mode between marrubiin and CTSC, molecular docking was performed in this study using the computer-aided drug design software Molecular Operating Environment (MOE) (2022.02). It should be specifically noted that the following docking results are theoretical predictions and have not yet been validated by experiments. The docking result predicted that the overall structure of marrubiin could be embedded into the active pocket of CTSC (Figure 6A). This active pocket is a hydrophobic cleft formed by the heavy chain and light chain of CTSC, and its spatial structure exhibits potential compatibility with the molecular conformation of marrubiin. Further docking result predicted that the lactone moiety of marrubiin might undergo a hydrolysis reaction, and then form a covalent bond with Cys234, a key functional residue of CTSC, as a potential covalent warhead (Figure 6B).

### 2.6. Marrubiin Suppresses Intracellular NSPs Activities and Protein Levels

**Figure 7:**
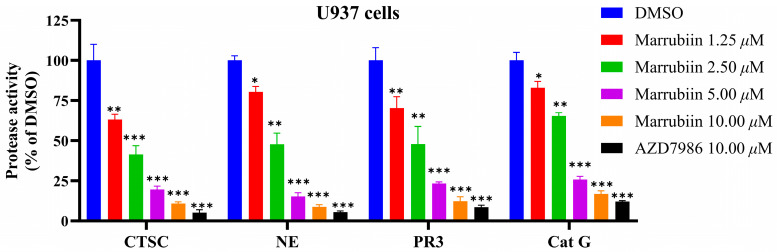
The effects of marrubiin on CTSC and NSPs activities in cells. Each group of the aforementioned experiments was independently repeated at least three times. GraphPad Prism 10.0 software was used to process and analyze the experimental data by means of One-way analysis of variance. Statistical significance was considered: * *p* < 0.05, ** *p* < 0.01 and *** *p* < 0.001 vs. DMSO group.

To evaluate the effect of marrubiin on downstream core biological indicators under in vitro conditions, the activity and expression of NSPs and CTSC were examined. The myeloid cell line U937 was used as a substitute for primary neutrophils. Cells were continuously treated with marrubiin working solution for 5 days, after which the activities of CTSC and NSPs were detected. The results showed that the activities of all proteases decreased to varying degrees (Figure 7). The inhibitory effect of marrubiin working solution on CTSC activity exhibited a significant dose dependence. Treatment with marrubiin working solution and AZD7986 working solution at the same concentration resulted in almost complete inhibition of CTSC activity. The changes in NSP activities showed the same trend as those of CTSC activity. At the highest concentration (10 μM), the activities of NE, PR3, and Cat G were inhibited to 8.2%, 10.3%, and 16.9% of those in DMSO group, respectively. Using the same assay protocol, effect of AZD7986 solution (10 μM) on NSP activities was detected, and the results showed that the activities of NE, PR3, and Cat G were inhibited to 5.1%, 8.1%, and 12.7%, respectively. These results indicated that although marrubiin may have multiple binding targets in cells, its inhibitory effects on activities of CTSC and NSPs are almost identical to those of synthetic small-molecule inhibitors.

**Figure 8:**
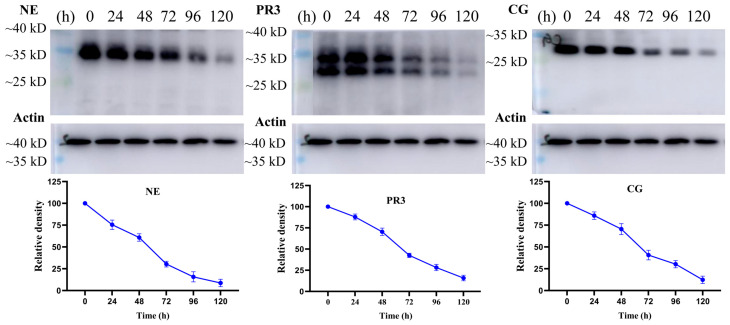
The effects of marrubiin on NSPs stability in U937 cells in 5 days. Each group of tests was independently repeated at least 3 times. GraphPad Prism 10.0 software was used to process and analyze the experimental data.

Next, the protein levels of NSPs in U937 cells after treatment with marrubiin for 5 days were evaluated using Western blot (Figure 8). After 24 h of treatment with 10 μM marrubiin, the content of NSPs in U937 cells remained at approximately 75.4–87.8% of the original level. However, after 48 h of treatment, the degradation trend of NSPs was significantly enhanced. After 96 h, NE, PR3, and Cat G were degraded to 15.7%, 28.2%, and 30.2% of original levels, respectively. Prolonging the treatment time ultimately resulted in the protein levels of NE, PR3, and Cat G being maintained at 8.7%, 15.8%, and 12.3% of original levels, respectively, rather than disappearing completely.

### 2.7. Marrubiin Suppresses CTSC and NSPs Activities In Vivo Without Detectable Toxicity

**Figure 9:**
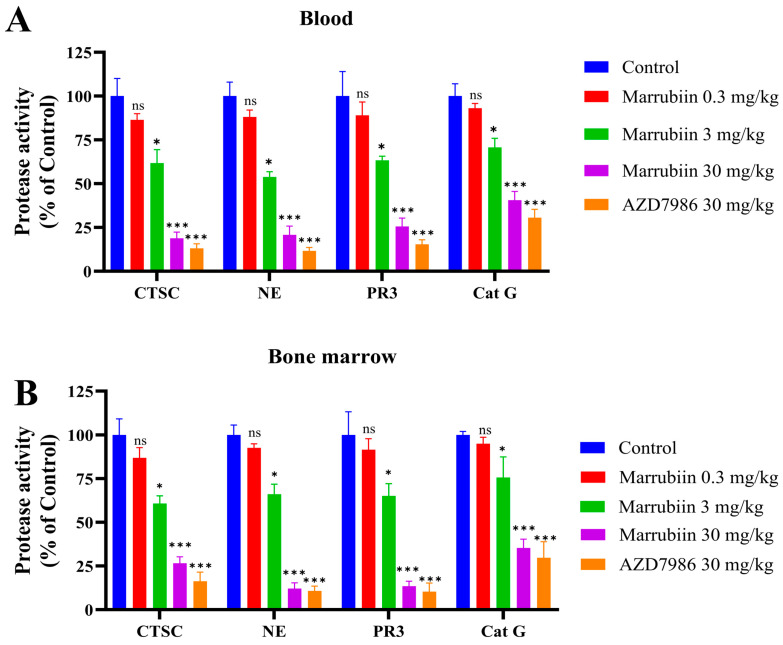
The activities of CTSC and NSPs in (**A**) blood and (**B**) bone marrow. *N* = 5 in each group. Each sample of test was independently repeated at least 3 times. GraphPad Prism 10.0 software was used to process and analyze the experimental data by mean of one-way analysis of variance. Statistical significance was considered: ns = no significant. * *p* < 0.05 and *** *p* < 0.001 vs. control group.

Following confirmation that marrubiin exerts inhibitory effects on NSPs activity by suppressing intracellular CTSC activity in vitro, further in vivo evaluations were conducted. C57BL/6 mice were administered marrubiin via daily intragastric gavage (at doses of 0.3, 3, and 30 mg/kg) for 7 consecutive days to assess its impact on the activities of CTSC and NSPs. The low-dose group (0.3 mg/kg) yielded suboptimal effects, with the activities of CTSC and NSPs in blood and bone marrow remaining between 80% and 90% of baseline levels. As the dose increased (3 mg/kg), the activities of CTSC and NSPs gradually decreased. In high-dose group (30 mg/kg), the remaining activity rates of CTSC, NE, PR3, and Cat G activities in bone marrow and blood were approximately 15.5%/25.5%, 20.9%/9.9%, 25.8%/13.6%, and 40.2%/35.5%, respectively (Figure 9). Notably, Cat G activities was less inhibited compared to NE and PR3, a phenomenon also observed in AZD7986-treated group (30 mg/kg), where remaining activity rates of CTSC, NE, PR3, and Cat G activities were approximately 10.6%/18.3%, 11.7%/8.1%, 15.8%/10.3, and 30.5%/35.2%, respectively [15].

**Figure 10:**
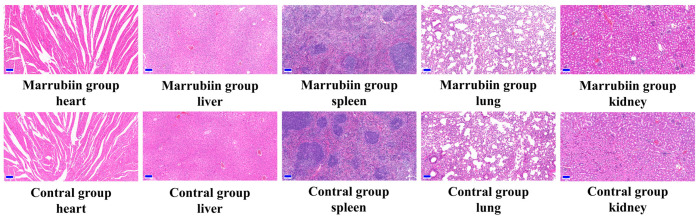
HE staining from heart, liver, spleen, lung, and kidney of mice. Scale bar: 100 μm.

To evaluate the safety of marrubiin, the body weight and toxic reactions of C57BL/6 mice in the high-dose administration group were recorded simultaneously. During the 7-day continuous administration period, no toxic reactions or significant weight loss were observed. No pathological changes were found in hematoxylin-eosin (H&E) staining of different tissues and organs (Figure 10). The above in vitro and in vivo experimental results collectively indicated that marrubiin is an effective CTSC inhibitor. While avoiding toxic, it can inhibit downstream NSPs activities and has the potential for treating inflammatory diseases.

### 2.8. Marrubiin Exerts Anti-Inflammatory Effects in AIA Model

**Figure 11:**
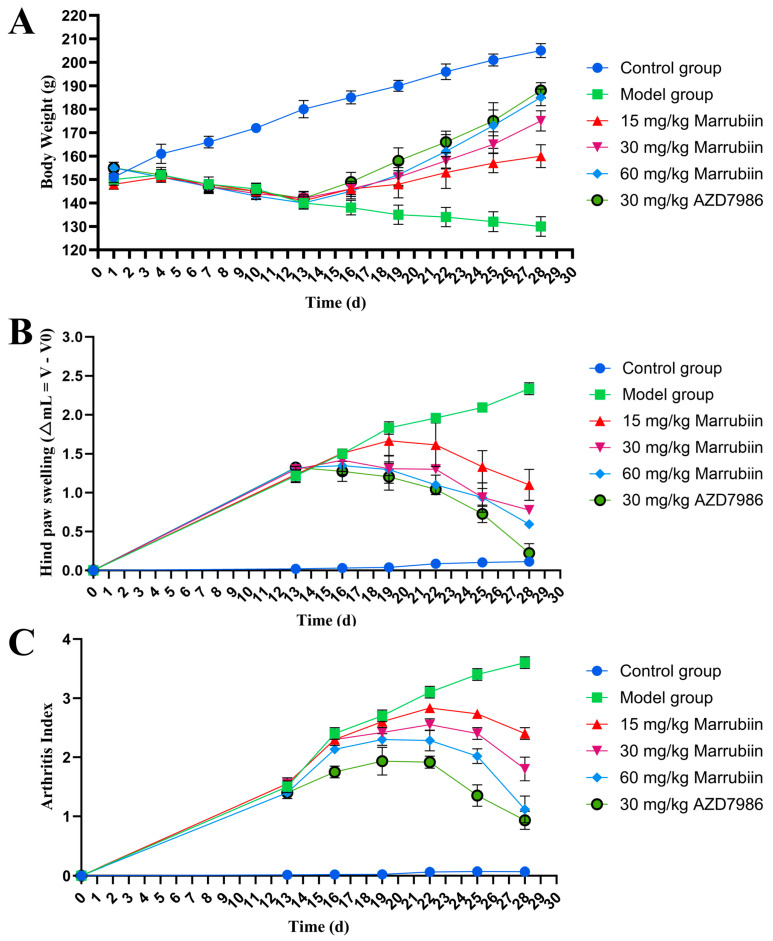
Marrubiin treatment alleviated disease severity and progression in rats with AIA. (**A**) Body weight changes in rats from different animal groups. (**B**,**C**) Measurement of hind paw swelling and arthritis index in rats at various time points. *N* = 5 in each group. GraphPad Prism 10.0 software was used to process and analyze the experimental data by means of one-way analysis of variance.

**Figure 12:**
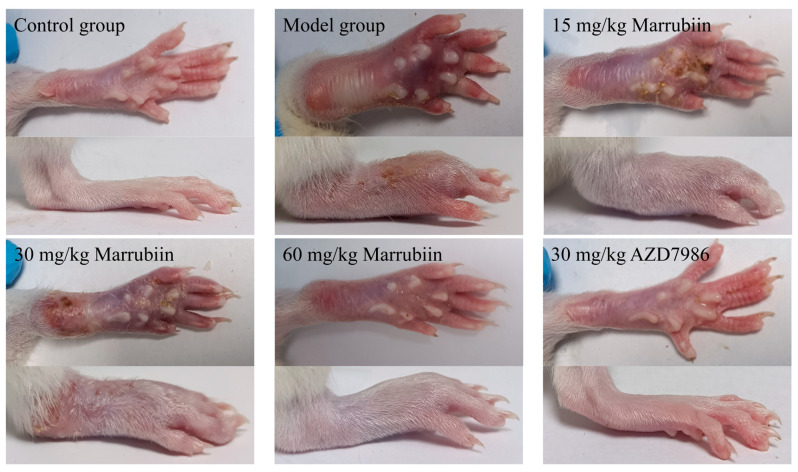
Representative images of right hind paws of rats in different groups.

The present study aimed to investigate the effect of marrubiin on disease progression in a rat AIA model. During the experiment, the body weight of rats was measured and recorded every 3 days starting from day 0. From day 13 onwards, the degree of paw swelling and arthritis scores were assessed and documented every 3 days. The results showed that compared with rats in normal group, those in model group and each compound-treated group exhibited varying degrees of body weight loss. From day 16 onwards, the body weight of rats in each treatment group began to recover gradually, with high-dose marrubiin group (60 mg/kg) and AZD-treated group (30 mg/kg) showing the most significant weight recovery effects (Figure 11A). In terms of paw inflammatory manifestations, rats in model group and each compound-treated group all presented with varying degrees of redness, swelling, and volume increase (Figure 12). However, compared with model group, treatment with different doses of marrubiin and AZD7986 all demonstrated therapeutic effects: between days 16 and 28, the inflamed paws of rats in the treatment groups showed a continuous reduction in volume and a gradual decrease in thickness (Figure 11B,C). The therapeutic effect of AZD7986 was particularly prominent, which was confirmed by the improved appearance of inflamed paws and clinical score results of rats. Although the therapeutic efficacy of marrubiin was slightly inferior to that of AZD7986, it still exhibited a significant therapeutic effect. Clinical score results indicated that from day 16 onwards, scores of 60 mg/kg marrubiin-treated group and 30 mg/kg AZD-treated group were significantly lower than those of AIA model group.

**Figure 13:**
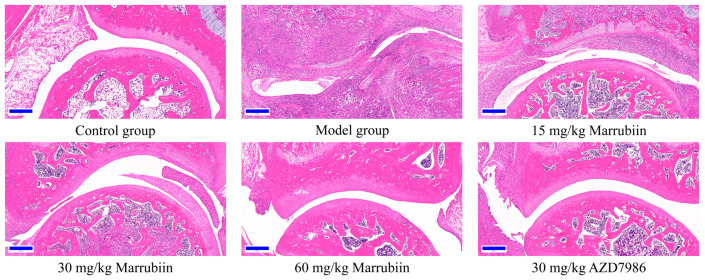
Exemplary micrographs of H&E-stained ankle joint sections. Scale bar: 200 μm.

**Figure 14:**
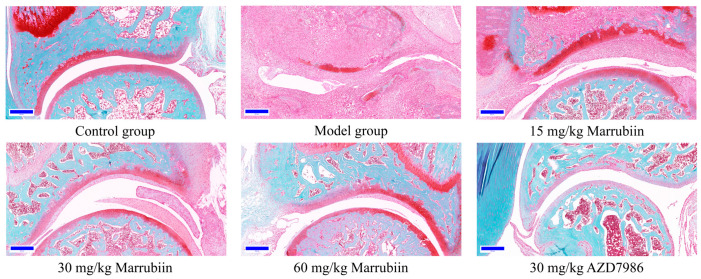
Exemplary micrographs of Safranin O/Fast Green-stained ankle joint sections. Scale bar: 200 μm.

To further elucidate the mechanism by which marrubiin improves disease scores, histopathological analysis of the arthritic joints was performed in this study. H&E staining was used to observe inflammatory cell infiltration, pannus formation, and synovial hyperplasia, so as to evaluate the degree of pathological damage caused by local joint inflammation. Safranin O/Fast Green staining was employed to assess the extent of cartilage damage. The results revealed that compared with animals in the healthy control group, all rats in the diseased groups exhibited histopathological features consistent with arthritis induction. Treatment with different doses of marrubiin and AZD7986 reduced the histopathological statement (Figure 13 and Figure 14).

**Figure 15:**
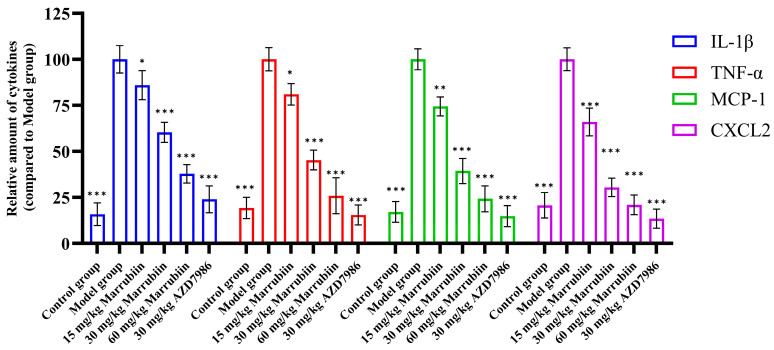
IL-1β, TNF-α, MCP-1, and CXCL2 levels in serum were measured using ELISA. *N* = 5 in each group. Each sample of test was independently repeated at least 3 times. GraphPad Prism 10.0 software was used to process and analyze the experimental data by means of One-way analysis of variance. Statistical significance was considered: * *p* < 0.05, ** *p* < 0.01 and *** *p* < 0.001 vs. Model group.

Additionally, the present study measured the levels of typical cytokines regulated by CTSC in serum, including IL-1β, TNF-α, MCP-1, and CXCL2. The results showed that abundance of cytokines changed in a concentration-dependent manner with increasing doses of marrubiin (Figure 15). Particularly in high-dose group, the expression levels of the aforementioned cytokines were significantly lower than those in model group and control group. Among them, magnitude of change in inflammatory factors in 60 mg/kg marrubiin-treated group was most similar to that in AZD7986-treated group.

**Figure 16:**
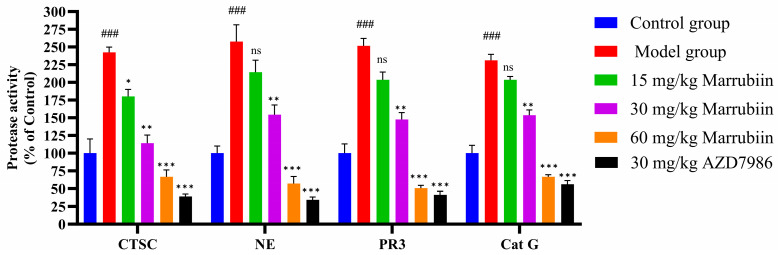
Efficiency study of marrubiin in vivo in AIA model. *N* = 5 in each group. Each sample of test was independently repeated at least 3 times. GraphPad Prism 10.0 software was used to process and analyze the experimental data by means of One-way analysis of variance. Statistical significance was considered: ns = no significant. ### *p* < 0.001 vs. Control group; * *p* < 0.05, ** *p* < 0.01 and *** *p* < 0.001 vs. Model group.

Finally, the activities of CTSC and NSPs in serum were detected in AIA model by inhibiting CTSC (Figure 16). The results demonstrated that compared with normal group, the activities of CTSC and NSPs in model group rats increased significantly after disease onset. After continuous drug treatment, the activities of both enzymes decreased to varying degrees. However, no significant reduction effect was observed in low-dose and medium-dose marrubiin-treated groups. Notably, the activities of CTSC and NSPs in high-dose marrubiin-treated group decreased significantly, with activities of CTSC, NE, PR3, and Cat G were reduced to approximately 58%, 47%, 50%, and 64% of those in control group, respectively. In contrast, AZD treatment restored activities of CTSC and NSPs to approximately 35%, 30%, 40%, and 58% of those in control group, respectively.

## 3. Discussion

Natural products, with their complex and sophisticated chemical structures as well as unique conformations and pharmacophores, enable effective binding to pharmacological targets, thereby exerting distinct biological activities [53]. Among their applications, natural products have the longest history in combating inflammatory diseases and have become an important source of lead compounds for drug development. CTSC is a novel and effective target that can be used for the treatment of various major inflammatory diseases such an IBD, COPD, and RA [10,11,12]. The screening and molecular modification of effective CTSC inhibitors, as well as their application in different diseases, are key tasks in the field of medicinal chemistry. However, an unavoidable issue is that synthetic CTSC inhibitors are confronted with problems such as metabolic instability, chemical instability, cytotoxicity, and intracellular inactivation [16,17,18]. Therefore, seeking inspiration from natural products is an ingenious approach to develop novel, highly effective, and safe CTSC inhibitors. These preliminary rational analyses have laid the foundation for this study.

To this end, a screening system for evaluating the inhibitory effect of compounds on CTSC enzymatic activity in vitro was established by our team, and activity assessment was conducted on 200 natural products. Prior to the implementation of this screening, confidence in obtaining effective CTSC inhibitors was relatively low. Given that the active site of CTSC is characterized by a deep and narrow binding pocket, a feature that may restrict the effective binding of natural products with large molecular weights or complex structures, we hypothesized that it would be difficult to identify active molecules through large-scale screening. Consistent with this prediction, most natural products failed to bind to CTSC effectively and exert inhibitory effects on its activity, primarily due to their complex chemical structures and large molecular weights. Among these compounds, protease inhibitor E-64 has been previously confirmed to exhibit weak inhibitory activity against CTSC, and in the current assay, its enzymatic inhibitory activity was measured at 15.0% [54]. This result demonstrated the validity and reliability of the large-scale screening conducted in this study.

Unexpectedly, five molecules were finally identified as effective CTSC inhibitors, and these molecules were scopine, acevaltrate, quillaic acid, L-cycloserine, and marrubiin, respectively. To comprehensively evaluate the enzymatic inhibitory properties of these five natural products, a systematic assay was performed using dose–response curves with 12 concentration points. Considering that compounds containing covalent groups may exhibit reduced intracellular activity and induce potential cytotoxicity due to their metabolic instability and high reactivity, CTSC inhibition assays based on cells and cytotoxicity evaluations were simultaneously conducted. Results from the concentration gradient assay showed that the activity differences observed in single-concentration assay were further amplified. Both scopine and acevaltrate are covalent inhibitors containing epoxypropane moiety, so they exhibit significant inhibitory activity against CTSC, although an approximate 10-fold difference in their inhibitory potency was observed (scopine CTSC Enz IC_50_ = 134.8 nM, acevaltrate CTSC Enz IC_50_ = 1053.2 nM). As a covalent structural unit, the epoxypropane moiety possesses the ability to bind to cysteine residues in a targeted manner, and the exertion of this ability is premised on the overall structure of the molecule being compatible with the active site of target. Structural differences between scopine and acevaltrate may lead to variations in their binding affinities for CTSC, which in turn affects the ability of their epoxypropane groups to bind to Cys234 in a covalent way. Scopine has a simpler chemical structure and an exposed epoxypropane group, which may be more conducive to stable binding with the active site of CTSC. Notably, both compounds have exhibited cellular activity, a finding that indicates they can bind effectively to CTSC under physiological conditions (scopine cell-based Enz IC_50_ = 134.8 nM, acevaltrate cell-based IC_50_ = 1053.2 nM). Quillaic acid, which has an aldehyde-terminated structure, exhibited potent inhibitory activity against CTSC in the cell-free system (quillaic acid CTSC Enz IC_50_ = 125.0 nM), but it failed to inhibit substrate degradation in intracellular assay. A possible explanation for this phenomenon is that the aldehyde group is an unstable chemical structure that may be rapidly oxidized in lysosomes [55], the organelle where CTSC is primarily localized, and this phenomenon has been reported previously [16,17]. Another compound that shows potent inhibitory activity against recombinant human CTSC but no intracellular activity is L-cycloserine. This compound has a relatively simple chemical structure characterized by a five-membered ring, and it may act directly on Cys234 of CTSC (L-cycloserine CTSC Enz IC_50_ = 1101.4 nM). However, its enzymatic inhibitory potency is limited due to the lack of additional structural units to further occupy the active pocket, and it cannot exert effects intracellularly. Furthermore, a notable observation is that D-cycloserine, the isomer of L-cycloserine, exhibits no enzymatic inhibitory activity at all. This result indicates that the configuration of molecule also affect inhibitory effect on CTSC activity, and this phenomenon has also been frequently reported in previous studies [56,57,58]. Different from the other four molecules, marrubiin does not exhibit obvious covalent warhead characteristic in its structure, and at least there is currently no clear evidence to support that its lactone structure can act as a covalent warhead. However, marrubiin was identified as the most promising CTSC inhibitor in this screening (marrubiin CTSC Enz IC_50_ = 57.5 nM).

Subsequent in vitro studies have preliminarily confirmed that marrubiin possesses anti-inflammatory activity, which is exerted by inhibiting the release of inflammatory mediator NO and reducing the levels of cytokines. Multiple research projects reported the pharmacological potential of marrubiin from various dimensions and emphasize its important role in anti-inflammation [46]. Of particular note to us is that other researchers found that marrubiin inhibits carrageenan-induced peritoneal inflammation by preventing inflammatory cell infiltration and peritoneal mast cell degranulation [47]. Regrettably, no clear results identified the specific target through which marrubiin exerts its anti-inflammatory effects. Therefore, in further studies, the effects of marrubiin on cellular CTSC and its downstream biological marker NSPs, were evaluated.

CETSA is a rapid, convenient, and highly reproducible method that has been widely used to verify the interaction between ligands and protein receptors [52]. CETSA results showed that marrubiin enhanced the thermal stability of CTSC in a dose-dependent manner, and this effect became more significant with increasing temperature, findings that indicate marrubiin can bind effectively to CTSC inside cells. The potential binding mode was predicted using MOE software, and the docking results revealed that marrubiin could embed into the hydrophobic active pocket of CTSC. On the basis of forming appropriate conformational adaptation with CTSC, marrubiin may form a covalent bond with Cys234 through its lactone structure, thereby stabilizing the interaction between the two. The above theoretical prediction results can provide reference directions for subsequent studies, such as clarifying the molecular origin of marrubiin’s biological activity and analyzing the potential affinity mechanism between marrubiin and CTSC. The binding characteristics of marrubiin to CTSC were preliminarily predicted through molecular docking calculations. However, such speculations still need to be further verified by subsequent experiments, including mass spectrometry analysis and time-dependent inhibition kinetics study, to confirm the binding mode and mechanism of action between marrubiin and CTSC.

The results of assays targeting intracellular CTSC and its downstream biological markers NSPs showed that the activity of all proteases decreased to varying degrees and exhibited a significant dose-dependent pattern. After cells were treated with marrubiin working solution and AZD7986 working solution at the same concentration, CTSC activity was almost completely inhibited, with less than 8% residual activity remaining. This phenomenon may be associated with the influence of CTSC-like enzymes. The change trend of NSPs activity was consistent with that of CTSC. Furthermore, when the activities of intracellular CTSC and NSPs were compared after treatment with marrubiin working solution and AZD7986 working solution at the same concentration, it was found that although marrubiin may have multiple binding targets in cells, its inhibitory effect on the activities of CTSC and NSPs was almost identical to that of the synthetic small-molecule inhibitor AZD7986. Long-term inhibition of CTSC activity leads to a gradual decrease in the level of NSPs, accompanied by the progressive degradation of NSP zymogens [7]. This process ultimately results in a reduction in NSP bands observed in Western blot experiments [19,20,21]. These phenomena have been repeatedly confirmed in both pharmacological inhibition experiments and gene knockout studies [7]. Similarly, in this study, Western blot analysis was used to detect the protein levels of NSPs in U937 cells 5 days after treatment with marrubiin. After treatment with 10 μM marrubiin for 5 days, the protein levels of NE, PR3, and Cat G were ultimately maintained at 8.7%, 15.8%, and 12.3% of their initial levels, respectively, and did not completely disappear.

Finally, in vivo studies were conducted to evaluate the effects of marrubiin on the activities of CTSC and NSPs, as well as its efficacy in animal models. Continuous administration of different doses of marrubiin to normal C57BL/6 mice exerted a significant dose-dependent effect on the activities of CTSC and NSPs in blood and bone marrow. It should be acknowledged that lower doses (0.3 and 3 mg/kg) of marrubiin failed to induce a significant inhibition of CTSC and NSPs activities. However, at high doses (30 mg/kg), the inhibitory effect exerted by marrubiin was almost comparable to that of AZD7986 (30 mg/kg). Notably, this effective dose is the one most frequently used in preclinical studies of AZD7986, and no obvious toxic side effects were observed in mice at this dose [59,60,61]. These results have strengthened our confidence in further conducting studies using animal disease models.

Theinactivated *Mycobacterium tuberculosis* component contained in Complete Freund’s Adjuvant (CFA), when injected in vivo, can trigger an excessive immune response in the body. This leads the immune system to erroneously attack the body’s own joint tissues, thereby inducing chronic inflammation. This process is accompanied by pathological changes such as synovial hyperplasia of the joints, inflammatory cell infiltration, and cartilage and bone destruction—symptoms that are highly similar to those of human RA. Therefore, the AIA model has been widely used for the in vivo evaluation of therapeutic drugs for autoimmune inflammatory diseases [62]. In addition, the potential of CTSC as a therapeutic target has been evaluated in various arthritis models [7,63,64,65]. The gross pathological changes and volume changes of rat hind paws clearly demonstrated that marrubiin exerted therapeutic effects at different levels under different administration doses, and its therapeutic effect at the high dose (60 mg/kg) was almost comparable to that of AZD7986 (30 mg/kg). The staining results of ankle joint section and the changes in levels of inflammatory factors in blood further confirmed that the therapeutic effect of marrubiin in high-dose group was comparable to that of AZD7986. Finally, the most important assessment, the activity assay of CTSC and NSPs in the blood, reflected again that although there was a difference in inhibitory effects on CTSC and NSPs between marrubiin and AZD7986, this difference was not as significant as we had expected. Based on the above experimental results, it can be inferred that the mechanism by which marrubiin exerts anti-inflammatory therapeutic effects in AIA model is closely related to the inhibition of CTSC activity. However, the possibility that marrubiin may also exert anti-inflammatory effects through other targets cannot be ruled out.

Our study reveals that marrubiin may exert anti-inflammatory effects by targeting and inhibiting CTSC. This result is consistent with the previously observed phenomenon that marrubiin prevents inflammatory cell infiltration and peritoneal mast cell degranulation [47].

## 4. Conclusions

In this study, we systematically identified and characterized marrubiin, a bioactive natural lactone isolated from *Marrubium vulgare*, as a potent and selective CTSC inhibitor with therapeutic potential for inflammatory diseases. Through large-scale screening and multi-step validation, marrubiin emerged as the optimal candidate, exhibiting strong inhibitory activity against both recombinant human CTSC (IC_50_ = 57.5 nM) and intracellular CTSC (IC_50_ = 51.6 nM) with minimal cytotoxicity. CETSA confirmed that marrubiin binds to CTSC, enhancing the thermal stability of CTSC and thereby suppressing its enzymatic activity. This inhibition directly impedes the maturation of downstream NSPs, as evidenced by reduced NSP activity and protein levels in vitro. Consistently, in vivo studies demonstrated that high-dose marrubiin (30 mg/kg) significantly reduced CTSC and NSP activities in blood and bone marrow, mirroring the efficacy of the clinical candidate AZD7986 (30 mg/kg). Functionally, marrubiin exerted anti-inflammatory effects by modulating cytokine networks. In LPS-stimulated RAW264.7 cells, it dose-dependently inhibited the release of NO and reduced the levels of pro-inflammatory cytokines (TNF-α, IL-1β, IL-6) and chemokines (MCP-1, CXCL2). In AIA model, marrubiin could alleviate disease symptoms, reduce redness and swelling of inflamed paws, improve histopathological damage, regulate the levels of inflammatory factors and chemokines, and effectively inhibit activity levels of CTSC and NSPs in serum. The predicted binding mode of marrubiin with CTSC showed that overall structure of marrubiin fits well into the active pocket of CTSC, and its lactone structure acts as a covalent warhead to form a covalent bond with the key residue Cys234. This action mechanism may be the origin of marrubiin’s biological activity and molecular basis for its affinity with target protein. Collectively, our findings demonstrate that marrubiin acts as a natural, effective CTSC inhibitor that suppresses NSP maturation and regulates inflammatory cascades, offering a promising therapeutic strategy for neutrophil-mediated inflammatory diseases.

## 5. Materials and Methods

### 5.1. Natural Product Information

The 200 compounds used for enzymatic activity assay are all unmodified natural products, which exhibit diverse biological activities and can be directly purchased from biological reagent company MedChemExpress (Monmouth Junction, NJ, USA). Detailed information regarding the representative natural products confirmed to possess CTSC inhibitory activity is as follows: scopine (HY-B0459, CAS 498-45-3, purity = 98.0%), quillaic acid (HY-N0839, CAS 631-01-6, purity = 99.30%), l-cycloserine (HY-B1122, CAS 339-72-0, purity = 99.44%), d-cycloserine (HY-B0030, CAS 68-41-7, purity = 98.07%), marrubiin (HY-6995, CAS 465-92-9, purity = 99.75%), acevaltrate (HY-N2070, CAS 25161-41-5, purity = 99.56%).

### 5.2. In Vitro CTSC Enzyme Assay

Both recombinant CTSL and CTSC for in vitro cell-free-based enzymatic activity assay can be purchased from R&D Systems (Minneapolis, MN, USA), a biotechnology company. According to the standardized procedures and material ratios provided by R&D Systems, prepare the activation solution (25 mM MES + 5 mM DTT, pH 6.0) and the assay solution (25 mM MES + 50 mM NaCl + 5 mM DTT, pH 6.0) in advance for later use. Dilute the detection substrate H-Gly-Arg-AMC to a 200 µM solution with the assay solution for standby. Prepare the working solution of compounds to be tested using the assay solution, with the concentration of working solution being 10 times the final test concentration. Dilute CTSC and CTSL, respectively, with cold activation solution. Thoroughly mix the two activation solutions containing the proteins to achieve final concentrations of 300 nM for CTSL and 440 nM for CTSC. After co-incubation for 1 h, the activated CTSC treated with CTSL can be used for the enzymatic activity inhibition efficacy test of compounds. Dilute the activated CTSC to 4.4 nM with cold assay buffer. Incubate 4 μL of the activated CTSC with 1 μL of the compound working solution (test group) or 1 μL of blank assay solution (control group) at room temperature for 3 h. Then, add 5 μL of the detection substrate to achieve a final concentration of 100 μM. Mix 5 µL each of the assay solution and the substrate solution as the blank group. After 1 h, the absorbance of detection substrate was measured using a multifunctional microplate reader at excitation/emission wavelengths of 380 nm/460 nm. The inhibitory efficacy of molecules on enzyme activity was calculated based on the absorbance difference between test group and control group. Following the above standard operating procedure, during the initial primary screening, all compounds were prepared into working solutions at 25 μM for enzyme inhibitory activity testing, and the enzyme activity inhibitory efficacy of compounds at a concentration of 2.5 μM was obtained. Further, molecules with an inhibitory efficacy of over 20% from the first-round screening were prepared into working solutions at 25 μM, 50 μM, and 100 μM for a 3-point gradient test of enzyme inhibitory activity. Finally, the top 5 performing molecules were prepared into working solutions at 0.048 μM, 0.097 μM, 0.195 μM, 0.391 μM, 0.781 μM, 1.562 μM, 3.125 μM, 6.25 μM, 12.5 μM, 25 μM, 50 μM, and 100 μM for a 12-point gradient test of enzyme inhibitory activity. Each group of tests mentioned above was independently repeated at least 3 times, and IC_50_ values were calculated using SPSS 10.0.

### 5.3. Cell Culture

The human-derived U937 cells and the mouse-derived RAW264.7 cells used in this study were purchased from the Cell Resource Center of the Shanghai Institute for Biological Sciences, Chinese Academy of Sciences. U937 cells were cultured in Roswell Park memorial institute 1640 medium supplemented with 10% fetal bovine serum, 100 U/mL penicillin, and 100 μg/mL streptomycin. RAW264.7 cells were maintained in high-glucose Dulbecco’s modified eagle medium containing 10% fetal bovine serum, 100 U/mL penicillin, and 100 μg/mL streptomycin. Both cell lines were incubated in a incubator at 37 °C with 5% CO_2_ and saturated humidity. The cells were passaged every 2–3 days, and those in the logarithmic growth phase were used for the experiments.

### 5.4. Intracellular CTSC Enzymatic Activity Assay

Cell-based CTSC enzymatic activity assays were performed in 96-well clear-bottom black microplates. Prepare the working solution of test compounds using serum-free medium as the solvent, with the concentration of working solution being 5 times the final test concentration (0.048 μM, 0.097 μM, 0.195 μM, 0.391 μM, 0.781 μM, 1.562 μM, 3.125 μM, 6.25 μM, 12.5 μM, and 25 μM). The detection substrate, H-Gly-Arg-AFC, was diluted to 100 µM in serum-free medium and stored for later use. U937 cells in the logarithmic growth phase were harvested, washed with PBS, and counted. A single-cell suspension was prepared at a density of 1.0 × 10^6^ cells/mL using serum-free medium. 30 µL of cell suspension was seeded into each well of the 96-well plate. To the test wells, 10 µL of the compound working solution was added, while 10 µL of serum-free medium was added to the control wells. The plate was pre-incubated in a cell culture incubator at 37 °C for 3 h. Subsequently, 10 µL of H-Gly-Arg-AFC was added to initiate the enzymatic reaction, and the plate was further incubated at 37 °C for 1 h. Fluorescence measurements were performed using a multimode microplate reader at excitation/emission wavelengths of 400/505 nm. Each group of tests was independently repeated at least 3 times, and IC_50_ values were calculated using SPSS 10.0.

### 5.5. Cytotoxicity Assay of Compounds

RAW264.7 cells and U937 cells in the logarithmic growth phase were counted and diluted, and the concentrations of the single-cell suspensions of the two types of cells were adjusted to 5 × 10^4^ cells/mL and 3 × 10^4^ cells/mL, respectively. After thorough mixing, the cells were seeded into a 96-well plate, with 100 μL of cell suspension added to each well. Prepare the working solution of test compounds using the corresponding medium as the solvent, with the concentration of working solution being 2 times the final test concentration (2.5 μM, 5 μM, 10 μM, 20 μM, and 40 μM). Twenty-four hours after cell seeding, the compound working solutions were added to the compound test wells, and the same volume of medium was added to the negative control wells, followed by continuous incubation for another 24 h. Only the wells with the same volume but without cells served as the blank control group. The CellTiter-Lumi™ luminescent cell viability assay kit (Beyotime Biotechnology, Shanghai, China, C0065XL) was used to detect cell viability according to the standard operating procedures. Each group of tests was independently repeated at least 3 times, and IC_50_ values were calculated using SPSS 10.0.

### 5.6. Selectivity Evaluation

The protocol for evaluating the inhibitory potency of compounds against other cathepsins referred to the CTSC activity assay. Recombinant human CTSB, CTSL, CTSS and CTSK for testing were also purchased from R&D Systems Biotechnology Company. Recombinant human Cat G, NE, and PR3 For testing were purchased from MedChemExpress. A multi-functional microplate reader was used to measure the absorption of Z-Leu-Arg-AMC at excitation/emission wavelengths of 380/460 nm to assess the activity changes of CTSB and CTSL, while the absorption of MCA-Arg-Pro-Lys-Pro-Val-Glu-NVAL-Trp-Arg-Lys(DNP)-NH2 at 320/405 nm was measured to evaluate the activity changes of CTSS and CTSK. Different assay buffers were prepared for diluting samples and measuring the activities of specific NSPs: a buffer containing 50 mM Tris and 750 mM NaCl (pH 7.4) was used for NE and PR3; a buffer containing 50 mM Tris, 750 mM NaCl, 5 mM EDTA (pH 7.4) was used for Cat G; and a buffer containing 25 mM MES, 50 mM NaCl, 5 mM DTT (pH 6.0) was used for CTSC. Synthetic substrates were employed to assess the activities of NSPs and CTSC: H-Gly-Arg-AMC for CTSC, N-MeSuc-Ala-Ala-Pro-Val-pNA for NE, aminobenzoyl-Val-Ala-Asp-Cys-Ala-Asp-Gln-ethylenediamine 2,4-dinitrophenyl for PR3, and N-Suc-Ala-Ala-Pro-Phe-pNA for Cat G. Each group of tests was independently repeated at least 3 times, and IC_50_ values were calculated using SPSS 10.0.

### 5.7. Measurement of NO Production and Cytokine Production Assays

RAW264.7 cells in the logarithmic growth phase were counted and diluted to a concentration of 6 × 10^4^ cells/mL. After thorough mixing, the cell suspension was seeded into 48-well plates and incubated in a humidified atmosphere containing 5% CO_2_ at 37 °C. Following 24 h of incubation, the culture medium was carefully aspirated, and the cells were gently washed with phosphate-buffered saline. Subsequently, test compounds dissolved in fresh medium were added to the respective wells at gradient concentrations (1.25 μM, 2.5 μM, 5 μM, 10 μM, and 20 μM), while control wells received an equal volume of vehicle-containing medium. After 1 h of pre-incubation, 30 μL of lipopolysaccharide (1 μg/mL) was added to each well except for the blank control group, which remained unstimulated. Following an additional 24 h incubation under identical conditions, the culture supernatants were collected to assess NO production using the Griess reagent assay. Concentrations of pro-inflammatory cytokines and chemokines were determined using commercially available ELISA kits according to the protocols. Each group of tests was independently repeated at least 3 times, and IC_50_ values were calculated using SPSS 10.0.

### 5.8. Western Blot Analysis

The Western blot experiments in this study were performed as described below. Cells in the logarithmic growth phase were collected in appropriate numbers according to different experimental purposes. After being thoroughly washed with phosphate-buffered saline and centrifuged, the cell pellets were resuspended in phosphate-buffered saline to obtain single-cell suspensions. Cells were fully lysed using RIPA buffer (Beyotime, P0013B) supplemented with EDTA-free protease and phosphatase inhibitor (Abcam, ab201120). Total cellular protein solutions were obtained after centrifugation to remove cell debris. The isolated proteins were quantified using the BeyoBCA protein assay kit (Beyotime, P0399M) following the operational protocol. The quantified protein samples were thoroughly mixed with protein loading buffer, boiled on a dry bath for 5 min, and then cooled to room temperature for Western blot analysis. The samples were separated by 10% sodium dodecyl sulfate-polyacrylamide gel electrophoresis and transferred to polyvinylidene fluoride membranes (0.2 μm, MilliporeSigma, Burlington, MA, USA, ISEQ00010). After blocking with Tris-buffered saline with tween-20 containing 5% skim milk for 2 h, the blotted membranes were incubated with specific primary antibodies overnight at 4 °C. The primary antibodies used included: anti-PR3 (Abcam, Cambridge, UK, AB103632, dilute1:1000), anti-NE (ZenBio, Chengdu, China, 381631, dilute 1:800), anti-Cat G (Invitrogen, Carlsbad, CA, USA, 38675A79, dilute 1:800), and anti-CTSC (ZenBio, 161516, dilute 1:1000). On the following day, the thoroughly rinsed membranes were incubated with appropriate horseradish peroxidase-conjugated secondary antibodies (Promega Corporation, Madison, WI, USA, W4022 for mouse and W4011 for rabbit) for 1 h. Finally, enhanced chemiluminescent horseradish peroxidase substrate (Tanon Science & Technology Co., Ltd., Shanghai, China, 180-506) was used to visualize protein bands on a ChemiDoc XRS system (Bio-Rad Laboratories, Inc., Hercules, CA, USA), and the protein bands were quantified using Image Lab analysis software (Version 6.1) (Bio-Rad).

### 5.9. Cellular Thermal Shift Assay

U937 cells in the logarithmic growth phase were equally divided into three portions. Two of the portions were thoroughly mixed with DMSO solutions of the compound at different concentrations, respectively, while the remaining portion was thoroughly mixed with the same volume of DMSO. The three portions of cells were continuously cultured under the same conditions for routine incubation. After 2 h, the three portions of U937 cells were collected separately and thoroughly washed twice with phosphate-buffered saline. The three cell pellets obtained by centrifugation were resuspended in phosphate-buffered saline to prepare three single-cell suspensions, and each single-cell suspension was equally divided into 6 PCR tubes. The PCR tubes were heated at the set temperatures of 37 °C, 43 °C, 49 °C, 55 °C, 61 °C, and 67 °C for 5 min, respectively, and then allowed to cool naturally to room temperature. Total proteins from each portion of cells were extracted according to the above protocol, and the isolated proteins were quantified, followed by Western blot analysis. Each group of tests was independently repeated at least 3 times. GraphPad Prism 10.0 software was used to process and analyze the experimental data.

### 5.10. Molecule Docking

Molecular docking was carried out using the MOE software, which excels in precisely simulating covalent bond formation. Co-crystal complex of AZD5248 and CTSC (PDB ID: 4CDE) was selected for covalent docking calculation. Ligand and protein were 3Dized and preprocessed according to standard protocols. The intact Cys234 residue obtained after initialization was chosen as the reactive site. Finally, docking calculations were performed using the induced fit model within the covalent docking module, with operations utilizing default settings.

### 5.11. NSPs Activity Detection In Vitro

U937 cells in the logarithmic growth phase were collected and equally divided into six portions. Four of these portions were treated with DMSO solutions of the test compound at varying concentrations to achieve final concentrations of 1.25, 2.5, 5, and 10 μM, respectively. An equal volume of DMSO was added to one portion as a blank control, while another portion treated with a DMSO solution of AZD7986 served as a positive control. The cells were cultured under standard U937 conditions for 5 consecutive days, with daily medium changes and fresh compound treatments administered using the same protocol. Following 5 days of treatment, cells were harvested, washed twice thoroughly with phosphate-buffered saline, and lysed in phosphate-buffered saline containing 1% Triton X-100. The lysates were centrifuged to collect the supernatant, and protein concentrations were determined as previously described to ensure equal total protein loading across all samples. The prepared samples were stored at −20 °C until further analysis. Different assay buffers were prepared for diluting samples and measuring the activities of specific NSPs: a buffer containing 50 mM Tris and 750 mM NaCl (pH 7.4) was used for NE and PR3; a buffer containing 50 mM Tris, 750 mM NaCl, 5 mM EDTA (pH 7.4) was used for Cat G; and a buffer containing 25 mM MES, 50 mM NaCl, 5 mM DTT (pH 6.0) was used for CTSC. Synthetic substrates were employed to assess the activities of NSPs and CTSC: H-Gly-Arg-AMC for CTSC, N-MeSuc-Ala-Ala-Pro-Val-pNA for NE, aminobenzoyl-Val-Ala-Asp-Cys-Ala-Asp-Gln-ethylenediamine 2,4-dinitrophenyl for PR3, and N-Suc-Ala-Ala-Pro-Phe-pNA for Cat G.

### 5.12. Stability of NSPs In Vitro

U937 cells in the logarithmic growth phase were treated with compound-containing DMSO solution or an equal volume of DMSO for 0, 1, 2, 3, 4, and 5 days. Cells were harvested at the same time each day and thoroughly washed twice with phosphate-buffered saline. The cell pellets obtained by centrifugation were resuspended in phosphate-buffered saline to prepare single-cell suspensions. Total protein solutions from cells collected each day were extracted and quantified following the aforementioned protocol, ensuring that the final total protein concentration of all samples was consistent. Western blot analysis was then performed as described above. Experimental data were processed and analyzed using GraphPad Prism 10.0 software to compare the relationship between the content of NSPs and the duration of compound treatment.

### 5.13. Animal Welfare

Experimental animals purchased from the Experimental Center of Anhui Medical University were housed in a controlled environment in accordance with the Guidelines on Animal Ethics and Welfare. The experimental procedures of this study were approved by the Ethics Committee of Anhui Medical University (LLSC20230868). All mice and rats were raised in SPF-grade animal rooms, with 5 mice or 3 rats per individually ventilated cage. The breeding environment was consistently maintained at a temperature of 20–26 °C, a humidity of 50–60%, a 12 h light/12 h dark cycle, and noise was controlled below 60 decibels. During the experiment, the animals were provided with regular chow and autoclaved distilled water. At the end of each experiment, for ethical considerations, the experimental mice were anesthetized with tribromoethanol and then sacrificed by cervical dislocation.

### 5.14. NSPs Activities and Safety Evaluation In Vivo

A total of 25 C57BL/6 mice (6–8 weeks old, weighing approximately 20 g, with equal numbers of males and females) were randomly divided into five groups. Mice in the compound-treated groups were orally administered the compound once daily at doses of 0.3, 3, and 30 mg/kg for 7 consecutive days. Mice in the positive control group were orally administered AZD7986 once daily at a dose of 30 mg/kg for 7 consecutive days. Mice in the normal group were given an equal volume of normal saline at the same time points. The body weight, death rate, and behavior were observed and recorded daily for one week. After 7 days, bone marrow and blood samples were collected from the mice and lysed using 8% Triton X-100 buffer. The lysates were stored at −20 °C until testing. The activity assays for CTSC and NSPs were performed as described above. The mice were anesthetized and the tissues were taken for H&E staining.

### 5.15. AIA Model Induction and Anti-Inflammatory Activity Evaluation of Marrubiin

Thirty 4-week-old male Sprague-Dawley rats (weighing 120–130 g) were randomly divided into 6 groups (5 rats per group) after 1 week of adaptive feeding, namely the normal group, model group, low-dose experimental group (15 mg/kg), medium-dose experimental group (30 mg/kg), high-dose experimental group (60 mg/kg), and positive control group (30 mg/kg). Starting from Day 0 of the experiment, rats in the administration groups and model group were injected intradermally with 0.1 mL of CFA into the left hind paw to induce AIA, while rats in the normal group were injected intradermally with an equal volume (0.1 mL) of normal saline as a control. After 13 consecutive days of feeding, rats treated with CFA successfully developed typical AIA symptoms. Subsequently, rats in the experimental groups were given different doses of the test compound via gavage for 15 consecutive days, and rats in the positive control group were given AZD7986 via gavage. During the experiment, the body weight, mental state, activity ability, hair color changes, and paw appearance changes of the rats were observed and recorded daily. In addition, on Day 0 of the experiment (baseline period), the basic paw volume of all rats was accurately measured using a plethysmometer, which served as a reference standard for subsequent evaluation of inflammation severity. From Day 13 to Day 28 of the experiment, the paw volume of the rats was measured repeatedly every 3 days. The degree of paw swelling was quantified by calculating the change in paw volume relative to the baseline. Meanwhile, the arthritis index score was calculated based on the range of paw redness and swelling, the severity of swelling, and the degree of limb movement limitation, so as to comprehensively evaluate the inflammatory state. On Day 28 of the experiment (the end of the administration cycle), blood samples were collected via abdominal aortic puncture. The blood samples were centrifuged at 3000 rpm for 20 min, and the upper serum was collected and stored at −20 °C for subsequent detection. The rats were then sacrificed, and their ankle joint tissues were harvested for subsequent pathological section observation and analysis. The cytokine levels in serum were measured. The activity detection of CTSC and NSPs was carried out according to the above methods.

### 5.16. Statistical Analysis

Statistical analyses were performed using SPSS software, while graphical data were generated with GraphPad Prism. The results of the in vitro tests were expressed as shown as means ± standard error of the mean (SEM). A *p*-value of less than 0.05 was considered statistically significant.

## Figures and Tables

**Table 1:** Activity evaluation of five natural products.

Cpd.	CTSC Enz (IC_50_: nM)	Cell-Based Enz (IC_50_: nM)	U937 Cytotoxicity (IC_50_: μM)	RAW264.7 Cytotoxicity (IC_50_: μM)
Marrubiin	57.5 ± 1.3	51.6 ± 3.2	>10	>10
Scopine	134.8 ± 4.7	337.0 ± 1.8	>10	>10
Quillaic acid	125.0 ± 2.9	>5000	>10	>10
Acevaltrate	1053.2 ± 2.2	702.1 ± 3.2	>10	>10
l-Cycloserine	1101.4 ± 2.0	>5000	>10	>10
AZD7986	9.0 ± 0.2	23.5 ± 1.2	>10	>10

Human recombinant CTSC isolated enzyme assay. CTSC cell-based assay using the U937 cell line. Cytotoxicity assay using the U937 cell line and RAW264.7 cell line. Each group of the aforementioned experiments was independently repeated at least three times, and IC_50_ values were calculated via logit regression analysis using SPSS 10.0.

**Table 2:** Cathepsin selectivity of marrubiin.

Cpd.	CTSC Enz (IC_50_: nM)	CTSL Enz (IC_50_: μM)	CTSS Enz (IC_50_: μM)	CTSB Enz (IC_50_: μM)	CTSK Enz (IC_50_: μM)
Marrubiin	57.5 ± 1.3	4.4 ± 0.5	>5	9.8 ± 1.1	>5

Human recombinant cathepsins isolated enzyme assay. Each group of the aforementioned experiments was independently repeated at least three times, and IC_50_ values were calculated via logit regression analysis using SPSS 10.0.

**Table 3:** Inhibitory effects of marrubiin on dipeptidyl peptidase.

Cpd.	Dipeptidyl Peptidase 4 Enz (IC_50_: μM)	Dipeptidyl Peptidase 8 Enz (IC_50_: μM)	Dipeptidyl Peptidase 9 Enz (IC_50_: μM)
Marrubiin	>10	>10	>10

Recombinant dipeptidyl peptidases isolated enzyme assay. Each group of the aforementioned experiments was independently repeated at least three times, and IC_50_ values were calculated via logit regression analysis using SPSS 10.0.

**Table 4:** Inhibitory effects of marrubiin on NSPs.

Cpd.	Cat G Enz (IC_50_: μM)	NE Enz (IC_50_: μM)	PR3 Enz (IC_50_: μM)
Marrubiin	>10	>10	>10

Recombinant NSPs isolated enzyme assay. Each group of the aforementioned experiments was independently repeated at least three times, and IC_50_ values were calculated via logit regression analysis using SPSS 10.0.

## Data Availability

The original contributions presented in the study are included in the article/Supplementary Materials, further inquiries can be directed at the corresponding author.

## Supplementary Materials

The following supporting information can be downloaded at: https://www.mdpi.com/article/10.3390/molecules30214170/s1, Figure S1: Results of three representative replicate experiments of CETSA; Figure S2: Results of three representative replicate experiments of NSPs stability; Figure S3: Images from more mice; Table S1: Primary screening; Table S2: 3 Conc Validation; Table S3: 12 Conc Validation.

## Abbreviations

The following abbreviations are used in this manuscript:

AIA	Adjuvant-induced arthritis
Cat G	Cathepsin G
CETSA	Cellular thermal shift assay
CFA	Complete Freund’s Adjuvant
COPD	Chronic obstructive pulmonary disease
CTSB	Cathepsin B
CTSC	Cathepsin C
CTSK	Cathepsin K
CTSL	Cathepsin L
CTSS	Cathepsin S
CXCL2	C-X-C motif chemokine ligand 2
DMSO	Dimethyl Sulfoxide
H&E	Hematoxylin-eosin
IBD	Inflammatory bowel disease
IL-1β	Interleukin-1 beta
IL-6	Interleukin-6
LPS	Iipopolysaccharide
MCP-1	Monocyte Chemoattractant Protein-1
NCFBE	Non-Cystic Fibrosis Bronchiectasis
NE	Neutrophil Elastase
NO	Nitric Oxide
NSP	Neutrophil Serine Protease
PR3	Proteinase 3
RA	Rheumatoid Arthritis
TNF-α	Tumor Necrosis Factor-alpha
