# Supplementary material for: Identification of Marrubiin as a Cathepsin C Inhibitor for Treating Rheumatoid Arthritis
Source: Molecules. 2025 Oct 23;30(21):4170. doi: 10.3390/molecules30214170 (PMC12610274; doi:10.3390/molecules30214170)
Supplement: Supplementary file 1 [file molecules-30-04170-s001.zip › Molecules SI.doc]

Identification of Marrubiin as a Cathepsin C Inhibitor for Treating Rheumatoid Arthritis

Feilong Zhou *^a,b 1^*, Yu Zhang *^b 1^*, Cui Chang *^b^*, Daxing Shi *^b^*,

Xing Chen *^b,*^*, Xinhua Liu *^b,*^*, Xiaobao Shen *^a,*^*

*^a^* *Medical School, Fuyang Normal University, Fuyang, 236037, P. R. China.*

*^b^ School of Pharmacy, Inflammation and Immune Mediated Diseases Laboratory of Anhui Province, Anhui Medical University, Hefei, 230032, P. R. China*

**CONTENT**

Western blot analysis results……………………………………………………............…2

Images from more mice…………………….......................……………………............…3

**Figure 1.** Results of three representative replicate experiments of CETSA.

**Figure 2.** Results of three representative replicate experiments of NSPs stability.

**Figure 3:** Images from more mice.
